# Remedies for Alternating Diseases

**Published:** 1891-07

**Authors:** 


### REMEDIES FOR ALTERNATING DISEASES.

Nausea and pain in head: Scilla.
Rheumatism and gastric symptoms: Kali-bichromicum.
Mental and physical symptoms: Platina.
Hard hearing and dim vision: Cicuta.
Hard hearing and otorrhœa: Pulsatilla.
Eye troubles and pains in lower extremities: Kreosot.
Headache and oppression of chest: Glonoine.
Rheumatism and cardiac pains: Benzoic acid.
Vertigo and colic: Veratrum-alb.
Eruptions and asthma: Caladium, Rhus-tox.
Pain in chest and abdomen: Æsculus-hip.
Headache and pain in abdomen: Cina, Plumbum.
Coughing and gaping: Antimon-tartar.
Constriction in chest and pain in abdomen: Calcarea-carb.
Headache and prolapsus recti: Arnica.
Pain in right temple and right knee: Melilotus.
Headache and backache: Melilotus.
Depression and good spirits: Actæa racemosa.
Spasm of glottis with contraction of fingers and toes: Asafœt.
Pain in forehead with crampy pains in chest: Lachnanthes.
Metrorrhagia and difficult breathing: Fluoric acid.
Eczema and internal affections: Graphites.
Herpes and dysentery: Rhus-tox.
Palpitations and pains in lower extremities: Benzoic acid.
Laryngeal and uterine complaints: Argentum-nitricum.
Epistaxis and hæmoptœ: Ferrum.
Delirium and colic: Plumbum.
Lumbago and headache: Aloes.
Lumbago and hemorrhoids: Aloes.
Eructations and gaping: Lycopodium.
Rheumatic and respiratory troubles: Guaiacum.
Diarrhœa and headache: Podophyllum.
Diarrhœa and rheumatism: Dulcamara.
Hæmoptœ and rheumatism: Ledum.
Fistula ani and chest troubles: Berberis, Calcarea-phosph.

Headache and gastralgia : Bismuth.

Alternation of sides : Lac-caninum.

Sore throat and sore eyes : Paris-quad.

Headache and uterine and abdominal affections : Aloes.

Asthma with headache : Angustura, Glonine, Kali-brom.

Constipation and diarrhœa : Ammon-mur., Sulphur.

Constipation alternating with diarrhœa :

I. Ammon-mur., Bry., Chel., Cimicif., Coffea, Cupr., Ign., Iod., Kobalt., Lach., Natr-mur., Nux-vom., Plumb., Pod., Puls., Sang.

II. Ant-crud., Arg-nitr., Ars., Kali-bichr., Mez., Op., perhaps Cocculus.

Asthma with nocturnal diarrhœa : Kali-carb.

Asthma with gout : Lycopodium.

Skin affections and pains in joints : Staphisagria.

Let your readers fill up all omissions, there are lots to be found yet in the materia medica. S. L.

